# Analysis of Genotype and Expression of FTO and ALKBH5 in a MENA-Region Renal Cell Carcinoma Cohort

**DOI:** 10.3390/cancers17091395

**Published:** 2025-04-22

**Authors:** Muna Abdalla Alhammadi, Burcu Yener Ilce, Poorna Manasa Bhamidimarri, Amal Bouzid, Nival Ali, Reem Sami Alhamidi, Alaa Mohamed Hamad, Mona Mahfood, Abdelaziz Tlili, Iman M. Talaat, Rifat Hamoudi

**Affiliations:** 1 Research Institute of Medical and Health Sciences, University of Sharjah, Sharjah P.O. Box 27272, United Arab Emirates; U21107591@sharjah.ac.ae (M.A.A.); bilce@sharjah.ac.ae (B.Y.I.); poorna.manasa@gmail.com (P.M.B.); abouzid@sharjah.ac.ae (A.B.); nali@sharjah.ac.ae (N.A.); ralhamidi@sharjah.ac.ae (R.S.A.); alaa.mohammed@sharjah.ac.ae (A.M.H.); 2 Clinical Sciences Department, College of Medicine, University of Sharjah, Sharjah P.O. Box 27272, United Arab Emirates; 3 Department of Biomedical Sciences, College of Health Sciences, Abu Dhabi University, Abu Dhabi P.O. Box 59911, United Arab Emirates; 4 Department of Applied Biology, College of Sciences, University of Sharjah, Sharjah P.O. Box 27272, United Arab Emirates; mmahfood@sharjah.ac.ae (M.M.); atlili@sharjah.ac.ae (A.T.); 5 Human Genetics and Stem Cell Research Group, Research Institute of Sciences and Engineering, University of Sharjah, Sharjah P.O. Box 27272, United Arab Emirates; 6 Pathology Department, Faculty of Medicine, Alexandria University, Alexandria 21131, Egypt; 7 Division of Surgery and Interventional Science, University College London, London NW3 2PS, UK; 8 Center of Excellence for Precision Medicine, University of Sharjah, Sharjah P.O. Box 27272, United Arab Emirates; 9 Biomedically Informed Artificial Intelligence Laboratory (BIMAI-Lab), University of Sharjah, Sharjah P.O. Box 27272, United Arab Emirates

**Keywords:** renal cell carcinoma, FTO, ALKBH5, RNA-modifying proteins

## Abstract

**Background/Objectives**: RNA-modifying proteins play a crucial role in the progression of cancer. The fat mass and obesity-associated protein (FTO) and alkB homolog 5 RNA demethylase (ALKBH5) are RNA-demethylating proteins that have contrasting effects in renal cell carcinoma (RCC) among different populations. This research investigates the genotype and expression levels of FTO and ALKBH5 in RCC patients from the Middle East and Northern Africa (MENA) region. **Methods**: Formalin-fixed paraffin-embedded samples from the kidney biopsies of RCC patients and controls were examined using targeted DNA sequencing, whole transcriptome profiling, and immunohistochemistry. **Results**: Our findings show that the rs11075995T variant in FTO is associated with a heightened risk of clear-cell RCC (ccRCC). ALKBH5 and FTO protein expression were significantly lower in ccRCC and chromophobe RCC (chRCC) patients but not in papillary RCC (pRCC) patients. In ccRCC, transcriptomic data revealed a significant downregulation of FTO (log_2_FC = −5.2, *q* < 0.001) and ALKBH5 (log_2_FC = −4.7, *q* < 0.001) compared to controls. A significant negative correlation was found in ccRCC between FTO expression and T allele frequency in rs11075995, suggesting that FTO expression is affected. **Conclusions**: This is the first demonstration of the association of the dysregulated expression of FTO and ALKBH5 in ccRCC and chRCC patients from the MENA region. FTO variant rs11075995T increased the risk of ccRCC and was negatively associated with FTO protein expression.

## Simple Summary

RNA-modifying proteins play a key role in cancer progression. The fat mass and obesity-associated protein (FTO) and alkB homolog 5 (ALKBH5), two RNA demethylases, have opposing effects on renal cell carcinoma (RCC). This study investigates their genetic and expression profiles in RCC patients from the Middle East and Northern Africa (MENA) region. We found that the FTO rs11075995T variant is associated with an increased risk of clear-cell RCC (ccRCC). Both FTO and ALKBH5 levels were lower in ccRCC and chromophobe RCC (chRCC) patients compared to controls. We found a significant negative relationship in ccRCC between FTO levels and the frequency of the T allele in rs11075995. This suggests that the variant may affect FTO expression. This study is the first to show the dysregulated expression of FTO and ALKBH5 in ccRCC and chRCC patients from the MENA region.

## 1. Introduction

Kidney cancer is a urological malignancy with a global incidence of 434,840 cases and a mortality rate of 155,953 deaths in 2022 [1]. In the Middle East and Northern Africa (MENA) region, kidney cancer saw an incidence of 9099 cases and a mortality of 4357 deaths in 2022 [2]. The most prevalent type of kidney cancer is renal cell carcinoma (RCC), accounting for 90% of kidney cancer cases [3]. The 5-year relative survival rate significantly decreases at advanced stages of RCC, dropping to only 12% for patients at stage IV [4]. RCC comprises heterogeneous epithelial tumors, with 75% of cases diagnosed as clear-cell RCC (ccRCC), the most common subtype. Other common RCC subtypes include papillary RCC (pRCC) and chromophobe RCC (chRCC), which account for 15% and 5% of RCC cases, respectively [3].

Over the last decade, omics technologies have been crucial in identifying novel cancer biomarkers across various cellular aspects, including the genome, transcriptome, proteome, epigenome, and metabolome. The main goals of multi-omics data analysis are to classify biological samples, predict clinical outcomes, uncover molecular mechanisms, and discover early diagnostic, prognostic, and predictive biomarkers [5]. Numerous studies using multi-omics data have explored RCC. Genomics and transcriptomics at the single-cell level have highlighted the cellular heterogeneity of ccRCC based on specific transcriptional factors [6]. Multi-omics has been applied to formalin-fixed paraffin-embedded (FFPE) ccRCC samples, integrating proteomics, mRNA, and miRNA sequencing. Proteomics highlighted pathways related to oxidative stress, while mRNA data concentrated on immune system modulation. This integration of omics technologies has provided a broader understanding of the biological processes in ccRCC [7].

RNA-modifying proteins (RMPs) are proteins that modify RNA molecules post-transcriptionally. Over 170 types of modifications exist, with N6-methyladenosine (m6A) being the most abundant type [8]. RNA modifications can be added, removed, or recognized by various RMPs belonging to three categories: writers, erasers, and readers [9]. These modifications influence RNA stability, splicing, translation, and nuclear export. Epitranscriptomics is the field that studies RNA modifications and their roles in health and disease. Recently, researchers have investigated the role of epitranscriptomics in various diseases, including cardiovascular diseases [10], diabetes [11], arthritis [12], and cancers [9].

FTO and ALKBH5 are RMPs that belong to the ALKBH family. They demethylate nucleic acids through an oxidation reaction that relies on iron (II) and α-ketoglutarate [13]. Recent studies have highlighted the various roles of FTO and ALKBH5 in tumorigenesis and cancer progression. For instance, decreased FTO expression has been independently associated with worse survival in prostate cancer by regulating epithelial–mesenchymal transition (EMT) [14]. FTO was found to be upregulated in human primary and 5-fluorouracil-resistant colorectal cancer (CRC) patients. Furthermore, inhibiting FTO in CRC cells increased sensitivity to 5-fluorouracil in chemo-resistant CRC cells [15]. ALKBH5 was upregulated in primary non-small-cell lung cancer (NSCLC) tissues, positively correlating with macrophage infiltration and programmed death-ligand 1 (PD-L1) expression, suggesting that ALKBH5 enhances susceptibility to anti-PD-L1 therapy in NSCLC [16]. In pancreatic cancer, the loss of ALKBH5 was linked to poor clinicopathological outcomes. It inhibits pancreatic cancer cell growth by activating period circadian regulator 1 (*PER1*) [17].

In RCC, RMPs have been found to play essential roles in promoting cancer hallmarks [18]. FTO and ALKBH5 have been shown to have conflicting roles in ccRCC. Some studies reported their oncogenic roles in ccRCC [19,20,21,22], while others noted tumor-suppressive roles [23,24]. This heterogeneity may be due to differences in population origin and genetic background. Therefore, further investigations are needed to unravel their roles in RCC patients from diverse populations. Additionally, assessing the genetic variants of FTO and ALKBH5 in RCC is important. This study aims to investigate FTO and ALKBH5 in an RCC cohort from the MENA region using targeted DNA sequencing to screen for *FTO* and *ALKBH5* variants, as well as whole transcriptomic analysis and immunohistochemistry (IHC) to identify the expression patterns of FTO and ALKBH5 associated with RCC.

## 2. Materials and Methods

### 2.1. Clinical Specimens

This retrospective study used FFPE renal biopsies collected from the Faculty of Medicine at Alexandria University. Ethical approvals for this study were obtained from the University of Alexandria, Egypt (IRB No.: 0306337) and the University of Sharjah (REC-23-12-08-01-PG). FFPE biopsies of malignant tissues were taken from patients primarily diagnosed with RCC who underwent radical or partial nephrectomy. Patient samples included ccRCC (39 samples), chRCC (7 samples), and pRCC (8 samples) subtypes and were collected from 2012 to 2022 (Table 1). Additionally, 11 non-cancerous control renal FFPE samples were obtained from a normal portion of kidney tissues from normal individuals or patients diagnosed with inflammatory renal diseases (Table 2). All RCC and control tissues were assessed by a pathologist. All FFPE samples were stored at room temperature until further analysis. Supplementary Table S1 outlines the samples that were used in the following experiments.

### 2.2. DNA Extraction from FFPE Biopsies

DNA was extracted from five to eight sequential sections, at 3 μm, from the FFPE tissue biopsies of the cases mentioned in Table 1 and Table 2 using a QiaAmp DNA mini kit (Qiagen, Hilden, Germany) according to the manufacturer’s instructions. Briefly, FFPE curls were deparaffinized using xylene. Then, the pellet was washed with 100% ethanol to remove the xylene. The samples were dried until all ethanol was evaporated. Next, protein digestion was performed by adding 180 μL buffer ATL and 20 μL proteinase K, followed by incubation at 56 °C for 2.5 h. RNA clean-up was achieved by adding 4 μL of RNase A after the incubation. The digested samples were treated with AL buffer and 100% ethanol, then washed with wash buffers on the DNeasy Mini spin column following the kit’s instructions.

### 2.3. Targeted DNA Sequencing

Next-generation sequencing (NGS) was conducted to sequence common variants in *FTO* and *ALKBH5*. Extracted DNAs were quantified using Nanodrop^TM^ (Thermo Fisher Scientific, Waltham, MA, USA) and used for the targeted sequencing using a Fluidigm Access Array, as previously described [26]. Primers were designed to cover common variants in the *FTO* (NG_012969.2) and *ALKBH5* (OMIM ID: 613303) genes (Supplementary Table S2). The primers were linked to Fluidigm-specific tag sequences: CS1: ACACTGACGACATGGTTCTACA for the forward primer and CS2: TACGGTAGCAGAGACTTGGTCT for the reverse primer. Around 20 ng DNA was taken from each sample and was amplified with 10 µM of each tagged primer using the Fast Start High Fidelity master mix (Roche, Basel, Switzerland) with the following cycling conditions: 1 cycle at 95 °C for 10 min, two cycles at 95 °C for 15 s, 60 °C for 4 min, and 13 cycles at 95 °C for 15 s, 72 °C for 4 min. After this, the amplified products were purified using ExoSAP-IT (Invitrogen, Waltham, MA, USA) with the following conditions: 1 cycle at 37 °C for 15 min and one cycle at 80 °C for 15 min.

Following purification, the amplicons were amplified using tagged primers on the 48.48 Access Array integrated fluidic circuit (IFC), employing the FastStart High Fidelity Master Mix (Roche, Basel, Switzerland). PCR products were then extracted from the Fluidigm 48.48 IFC (Fluidigm Europe B.V., Amsterdam, The Netherlands).

After transferring the harvested products from each sample inlet, they underwent 100-fold dilution using nuclease-free water. After that, they were attached to the access array barcode library and underwent thermal cycling at the following conditions: 1 cycle at 95 °C for 10 min, 15 cycles at 95 °C for 30 s, 60 °C for 30 s, 72 °C for 1 min, and one cycle at 72 °C for 3 min. AMPure XP beads (Beckman Coulter, Brea, CA, USA) were used to purify the prepared amplicon library. A High Sensitivity DNA assay kit on a BioAnalyzer (Agilent, Santa Clara, CA, USA) was used to quantify the prepared library. After diluting the libraries to ~100 pM, they were sequenced on an Ion S5 XL Semiconductor sequencer using an Ion 520 Chip (Life Technologies Corporation, Carlsbad, CA, USA) prepared on a fully automated Ion Chef System (Thermo Fisher Scientific, Waltham, MA, USA).

### 2.4. DNA Sequencing Data Analysis

The DNA sequencing data were analyzed using an in-house bioinformatics pipeline, which included raw data processing, alignment to the reference genome hg19 (GRCh37), and a quality control assessment, as previously described [27]. Binary alignment map (BAM) files were visualized using Integrative Genomics Viewer (IGV) version 2.15.4 (Broad Institute, Cambridge, MA, USA) [28]. Depending on the percentage of the mutated allele, individuals were classified as homozygous or heterozygous. Individuals with a percentage of the mutated allele lower than 35% are identified as homozygous wild-type. Heterozygous genotypes were identified as those with a mutant allele percentage greater than or equal to 35% and less than 70%. Homozygous mutant individuals have an allele percentage greater than or equal to 70% [29].

### 2.5. Sanger Sequencing

To validate the rs11075995 variant detected by NGS for the *FTO* gene, Sanger sequencing was conducted on DNA extracted from a group of RCC samples and control samples from the same cohort that underwent NGS [30] using targeted sequencing primers (Supplementary Table S2). Samples demonstrating adequate DNA quality and quantity were selected for Sanger sequencing. PCR products were treated with the ExoSAP-IT PCR Product Cleanup Reagent (78200.200.UL, Applied Biosystems, Thermo Fisher Scientific, Waltham, MA, USA). Then, sequencing reactions were carried out using a BigDye Terminator v3.1 Cycle Sequencing Kit (4337455, Applied Biosystems, Thermo Fisher Scientific, Waltham, MA, USA). After that, purification and precipitation were conducted using the ethanol/EDTA/sodium acetate precipitation method. Capillary sequencing was performed using a Genetic Analyzer 3500 (Applied Biosystems, Thermo Fisher Scientific, Waltham, MA, USA). The sequences were analyzed using FinchTV version 1.4.0 (Geospiza, Inc., Seattle, WA, USA).

### 2.6. Immunohistochemistry

RCC and control FFPE tissue samples were stained to detect the expression of FTO and ALKBH5. The FFPE samples were sectioned at 4 μm. The sections were deparaffinized and rehydrated, followed by heat-mediated antigen retrieval with Tris/EDTA buffer at pH 9.0 for FTO and ALKBH5. Endogenous peroxidase was quenched, and nonspecific binding was blocked using a mouse- and rabbit-specific HRP/DAB (ABC) detection IHC kit (catalog number: ab64264, Abcam, Waltham, MA, USA). After that, the sections were stained with a recombinant anti-FTO antibody (Abcam, Waltham, MA, USA, catalog number: ab126605) at 1:500 dilution or a recombinant anti-ALKBH5 antibody (Abcam, Waltham, MA, USA, catalog number: ab195377) at 1:2200 dilution. The sections were kept overnight at 4 °C in a humidified chamber. After that, secondary antibody conjugation and 3,3′-Diaminobenzidine (DAB) staining were carried out. The slides were counter-stained with hematoxylin, dehydrated, and mounted.

The slides were observed and captured using an Olympus DP74 microscope digitally attached to a BX43 microscope (Olympus Life Sciences, Tokyo, Japan). The expression status was evaluated using the immunoreactive score (IRS), which was generated by multiplying the staining intensity (0: negative, 1: low, 2: moderate, and 3: high intensity) by the percentage of positively stained cells (0: no positive cells, 1: 1–25%, 2: 26–50%, 3: 51–75%, and 4: 76–100% positively stained cells), with a range of 0 to 12 [31,32]. Two independent investigators performed a microscopic evaluation of the slides. Interobserver variability was addressed by conducting consensus meetings between the two independent observers to resolve discrepancies. In instances where a consensus could not be reached, the average of both observers’ scores was used as the final value. Additionally, DAB staining was semi-quantitatively assessed using the IHC toolbox plugin in ImageJ version 1.52a (National Institutes of Health, Bethesda, MD, USA) [33]. Optical density (OD) was calculated as follows:

(1) $$OD=\frac{maximum\;intensity}{mean\;intensity}$$

### 2.7. RNA Extraction from FFPE Biopsies

For the samples that proceeded to RNA sequencing (RNA-seq) and quantitative real-time PCR (RT-qPCR), RNA was extracted from FFPE tissue following the protocol of the RecoverAll™ Total Nucleic Acid Isolation Kit (Invitrogen, Waltham, MA, USA) with some modifications. Briefly, eight to ten curls were cut from each sample at 3 μm. The FFPE curls were deparaffinized by xylene. After that, the pellet was washed with 100% ethanol. Then, the samples were dried until all ethanol was evaporated. After that, the steps of protein digestion, total nucleic acid isolation, and washing were conducted as per the kit’s instructions. After nucleic acid extraction, the TURBO DNA-free™ kit (Invitrogen, Waltham, MA, USA) was used to ensure the removal of genomic DNA from RNA samples.

### 2.8. RNA Sequencing

RNA samples from 13 ccRCC patients and 10 control samples were analyzed by whole-transcriptome sequencing using the AmpliSeq Whole Transcriptome on the S5 System (Thermo Fisher Scientific, Waltham, MA, USA), as previously described [34]. The targeted RNA-seq library was prepared using the Ion AmpliSeq Transcriptome Human Gene Expression Kit (Thermo Fisher Scientific, Waltham, MA, USA). After that, the library was purified using Agencourt AMPure XP Beads (Beckman Coulter, Indianapolis, IN, USA) and quantified using an Ion Library TaqMan™ Quantitation Kit (Applied Biosystems, Thermo Fisher Scientific, Waltham, MA, USA). After diluting the libraries to 100 pM, they were pooled equally. The resulting template libraries were then sequenced on the Ion S5 XL Semiconductor sequencer using an Ion 540 Chip (Life Technologies Corporation, Carlsbad, CA, USA) prepared on a fully automated Ion Chef System (Thermo Fisher Scientific, Waltham, MA, USA).

RNA-seq data were analyzed using Ion Torrent Software Suite version 5.4 (Thermo Fisher Scientific, Waltham, MA, USA). The alignment was carried out using the Torrent Mapping Alignment Program (TMAP) against the reference sequence derived from the hg19 (GRCh37) assembly. The transcriptome analysis was focused on *FTO* and *ALKBH5*.

### 2.9. Gene-Specific cDNA Synthesis and RT-qPCR

Gene-specific reverse transcription was performed for the following target genes: *FTO*, *ALKBH5*, and *HMBS*, using the SuperScript™ III First-Strand Synthesis System (Invitrogen, Waltham, MA, USA). In brief, 200 ng RNA was mixed with 1 μL of dNTP mix and 1 μL of a mixture of equimolar reverse primers. The volume was completed to 10 μL using nuclease-free water. The tubes were incubated at 65 °C for 5 min followed by incubation on ice for 2 min. Then, the following components were added to each sample: 2 μL of 10× RT buffer, 4 μL of MgCl_2_, 2 μL of DTT, 1 μL of RNaseOUT, and 1 μL of SuperScript III RT. The samples were incubated at 50 °C for 50 min, followed by 85 °C for 5 min and 2 min on ice. After that, 1 μL of *E. coli* RNase H was added, followed by incubation at 37 °C for 20 min. The reverse-transcribed samples were kept at −80 °C until use.

RT-qPCR of the reverse-transcribed samples by gene-specific cDNA synthesis was conducted on *FTO*, *ALKBH5*, and *HMBS*, which acted as a reference gene. The primers’ sequences and efficiencies are listed in Supplementary Table S3. The qPCR reaction was performed using the Maxima SYBR Green/ROX qPCR Master Mix (Thermo Fisher Scientific, Waltham, MA, USA) on a QuantStudio3 Real-Time PCR thermal cycler (Applied Biosystems, Thermo Fisher Scientific, Waltham, MA, USA). The relative expression levels of the target gene were calculated using the 2^−ΔΔCt^ method relative to the *HMBS* gene [35].

### 2.10. Retrieval of FTO and ALKBH5 Targets

The RM2Target database [36,37] was utilized to retrieve the targets of the FTO and ALKBH5 proteins. The list of targets was retrieved from RM2Target-validated data from the hg19(GRCH37) assembly, followed by selecting the targets of FTO or ALKBH5. The expression profiles of the targets were compared with the RNA-seq results of our cohort of ccRCC and control samples.

### 2.11. Statistical Analysis

For DNA sequencing analysis, odds ratios were calculated based on allele counts, and the significance was assessed using chi-square and Fisher’s exact tests using SPSS version 21.0.0.0 (IBM Corporation, Armonk, NY, USA). Haldane–Anscombe correction was performed if there were values of zero in the contingency tables. RNA-seq data were analyzed using the R package DESeq2 v.1.38.3 [38,39]. Significant differentially expressed genes (DEGs) were defined as genes with an absolute log_2_ fold change (FC) ≥ 1.5 and *q*-value < 0.05. The statistical software R v.4.2.1 [39] was used to generate boxplots using the ggpubr package (v.0.5.0) [40], correlation plots were generated using the corrr package (v.0.4.4) [41], and heatmaps were generated using the pheatmap package (v.1.0.12.) [42]. A Student’s *t*-test or analysis of variance (ANOVA) followed by the Tukey post hoc test was used for the determination of *p*-values (* *p* < 0.05, ** *p*  < 0.01, *** *p* < 0.001). They were conducted using the rstatix package (v.0.7.1) [43]. In the boxplots, the lower and upper hinges indicate the first and the third quartiles, respectively, with the median represented as the line across the box. The whiskers extend from the hinges to the smallest and largest values within the 1.5× interquartile range (IQR).

## 3. Results

### 3.1. The rs11075995T Variant in FTO Is Associated with an Increased Risk of ccRCC

**Figure 1:**
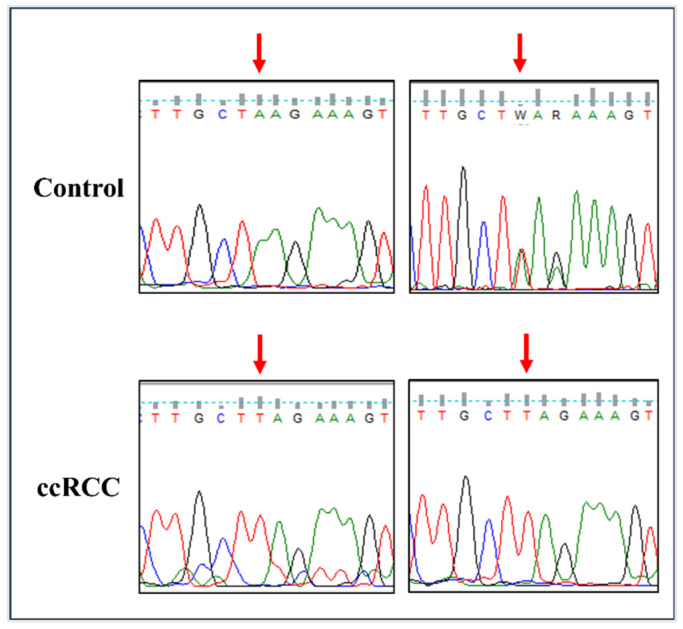
Sanger sequencing of the rs11075995 polymorphism in the *FTO* gene in selected control and ccRCC cases. Arrows indicate the position of the rs11075995 polymorphism. ccRCC, clear-cell renal cell carcinoma.

Targeted DNA sequencing was conducted on common *FTO* and ALKBH5 variants previously reported to be associated with cancer risk. ALKBH5 polymorphisms rs2047281425, rs61999283, and rs17855125 were similar to the reference genotypes (CC, GG, and CC, respectively) in all RCC cases and controls. The homozygous AA genotype was detected at the rs8068517 G>A variant in ALKBH5 in all tested cases and controls. Among the tested ccRCC patients, 14 patients were homozygous for *FTO* variant rs11075995T, seven were heterozygous, and one was homozygous with the reference allele (AA). However, in controls, four samples were TT homozygous, four samples were heterozygous, and three samples were homozygous for the reference allele (AA). The frequency of the rs11075995T allele was significantly associated with an increased risk of ccRCC (per-allele OR = 3.24, 95% CI; 1.06–9.87) compared to controls (Table 3). Due to its significance with ccRCC risk, the rs11075995 (A>T) polymorphism was validated on selected cases of ccRCC and controls using Sanger sequencing (Figure 1). The other tested *FTO* and ALKBH5 variants (rs1121980, rs17817449, rs9939609, rs8050136, and rs8400) did not show a significant difference between controls and RCC patients.

### 3.2. FTO and ALKBH5 Proteins Have Lower Expression in ccRCC and chRCC Patients

**Figure 2:**
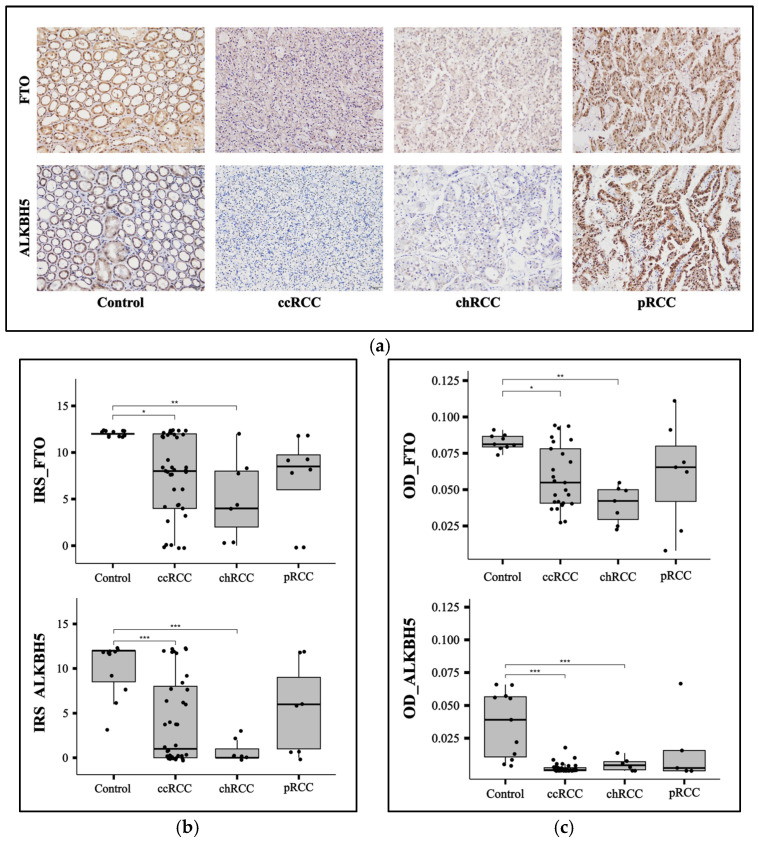
The protein levels of FTO and ALKBH5 were measured by IHC in a cohort including 11 controls and 39 ccRCC, 7 chRCC, and 8 pRCC patients. (**a**) Representative IHC images are presented. The scale bar represents 50 μm. IHC staining was analyzed using (**b**) IRS and (**c**) ImageJ. Asterisks represent the *p*-values of the ANOVA test followed by the Tukey post hoc test (* *p* < 0.05, ** *p* < 0.01, *** *p* < 0.001). FTO fat mass and obesity-associated protein, ALKBH5 alkB homolog 5 RNA demethylase, ccRCC, clear-cell renal cell carcinoma; chRCC, chromophobe renal cell carcinoma; pRCC, papillary renal cell carcinoma; IRS, immunoreactive score; OD, optical density; ANOVA, analysis of variance.

**Figure 3:**
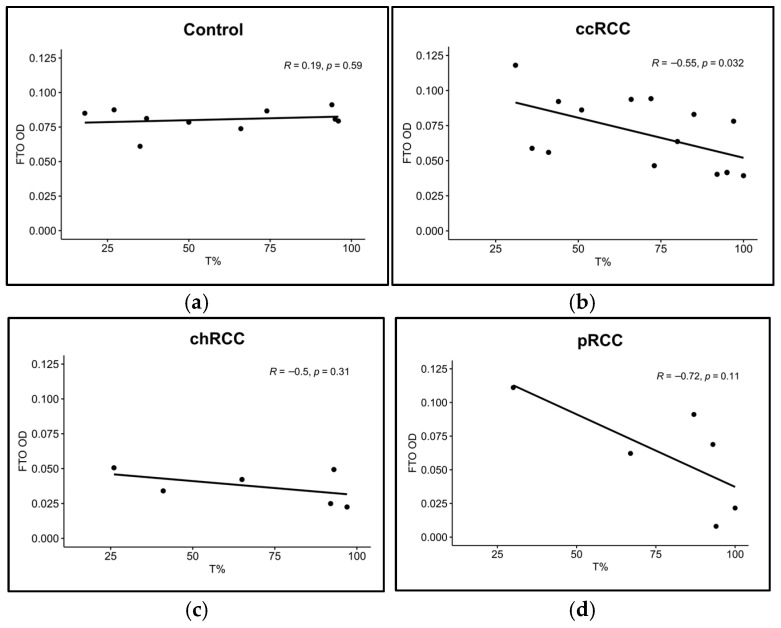
Correlation between FTO protein expression and the mutated allele frequency at rs11075995. Pearson correlation was conducted on (**a**) control (*n* = 10), (**b**) ccRCC (*n* = 15), (**c**) chRCC (*n* = 6), and (**d**) pRCC (*n* = 6) samples. ccRCC, clear-cell renal cell carcinoma; chRCC, chromophobe renal cell carcinoma; pRCC, papillary renal cell carcinoma; FTO, fat mass and obesity-associated protein; OD, optical density; *R*, Pearson correlation coefficient.

To evaluate the protein expression of FTO and ALKBH5 proteins in our RCC cohort relative to control samples, we performed IHC on FTO and ALKBH5 on ccRCC (*n* = 39), chRCC (*n* = 7), pRCC (*n* = 8), and control (*n* = 11) kidney FFPE samples (Figure 2a). IHC results were assessed using IRS (Figure 2b) and ImageJ (Figure 2c). ALKBH5 and FTO showed significantly lower expression in RCC patients with ccRCC (*p* < 0.001 for ALKBH5 and *p* < 0.05 for FTO) and chRCC (*p* < 0.001 for ALKBH5 and *p* < 0.01 for FTO) subtypes. However, there was no significant difference in the ALKBH5 and FTO levels in pRCC samples compared to controls (Figure 2b,c). To analyze the effect of the rs11075995T variant in the *FTO* gene that was detected by NGS in ccRCC patients compared to controls, a correlation plot was made between FTO protein expression and T allele frequency at the rs11075995 variant. A significant negative correlation was observed in ccRCC patients (R = −0.55, *p* = 0.032) (Figure 3b). However, the correlation was insignificant in controls and the other RCC subtypes (Figure 3a,c,d).

### 3.3. The Expression of FTO and ALKBH5 Genes Is Downregulated in ccRCC

**Figure 4:**
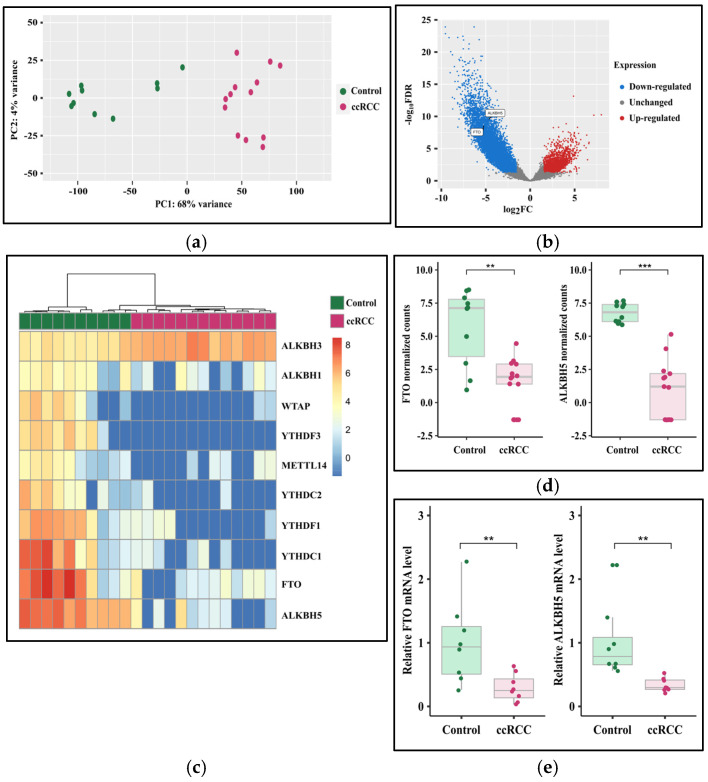
Downregulation of FTO and ALKBH5 genes in ccRCC patients compared to controls. RNA-seq of ccRCC patients (*n* = 13) and controls (*n* = 10) presented by (**a**) principal component analysis, (**b**) volcano plot, (**c**) heatmap of differentially expressed genes of RNA-modifying proteins, and (**d**) boxplot of normalized counts of FTO and ALKBH5. The results were validated by (**e**) RT-qPCR performed for ccRCC patients (*n* = 8) and controls (*n* = 8). Asterisks represent the *p*-values of the Student’s *t*-test (** *p* < 0.01, *** *p* < 0.001). RNA-seq, RNA sequencing; FTO, fat mass and obesity-associated protein; ALKBH5, alkB homolog 5 RNA demethylase; ccRCC, clear-cell renal cell carcinoma; PC, principal component; FDR, false discovery rate; FC, fold change.

Because ccRCC is the most prevalent RCC subtype, we assessed *FTO* and *ALKBH5* expression in ccRCC patient samples at the RNA level. Whole transcriptomic RNA-seq analysis revealed a significant difference between ccRCC patients and controls (Figure 4a,b). Principal component analysis (PCA) showed a clear separation between ccRCC patients and control samples, confirming the unique transcriptomic profile (Figure 4a). RNA-seq results showed that *FTO* and *ALKBH5* expression levels were significantly reduced in ccRCC patients (Figure 4b), and based on a log_2_FC of <−1.5 and >1.5 and a *q*-value < 0.05, 8640 genes were shown to be significantly downregulated and 2229 genes significantly upregulated in ccRCC patients. A significant downregulation of both *FTO* (log_2_FC = −5.2, *q*-value < 0.001) and *ALKBH5* (log_2_FC = −4.7, *q*-value < 0.001) genes were found in ccRCC relative to controls (Figure 4c,d). Downregulation of *FTO* (*p* < 0.01) and *ALKBH5* (*p* < 0.01) was confirmed using RT-qPCR on ccRCC patients (*n* = 8) relative to controls (*n* = 8) from our cohort (Figure 4e).

### 3.4. The Expression of FTO and ALKBH5 Target Genes

**Figure 5:**
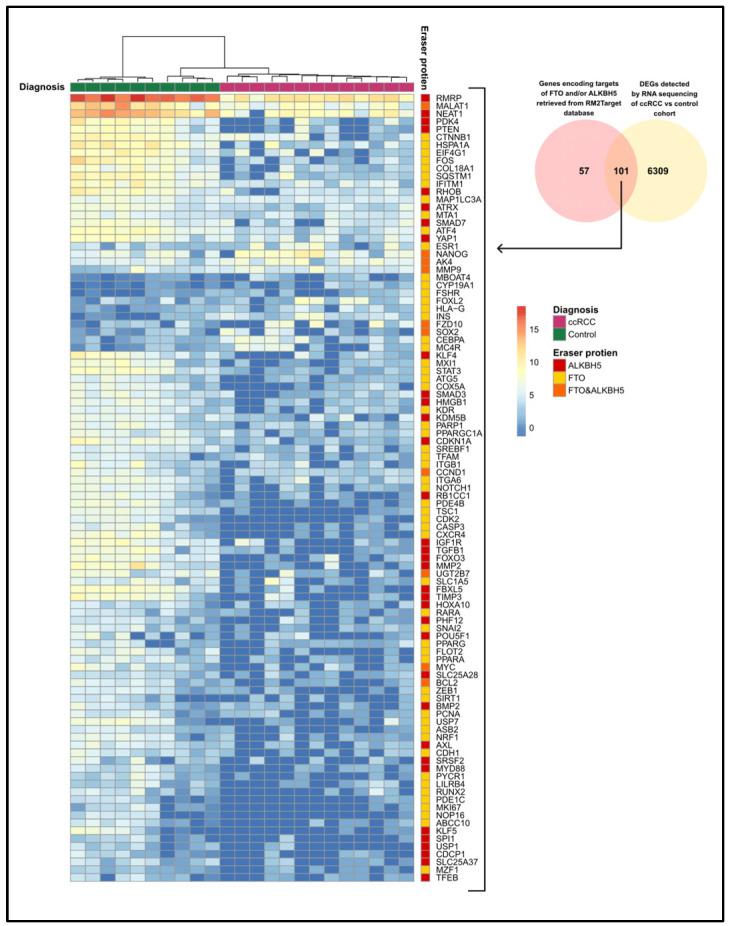
The expression of genes encoding the target RNAs of FTO and ALKBH5 proteins in the cohort of ccRCC patients (*n* = 13) and controls (*n* = 10). Genes encoding targets of FTO and/or ALKBH5 were retrieved from the RM2Target database and compared to differentially expressed genes detected by transcriptome profiling in our ccRCC and controls cohort. The eraser protein targeting each gene is specified beside each gene’s name. DEG, differentially expressed gene.

To analyze the expression levels of genes encoding the target RNAs of *FTO* and *ALKBH5*, we used the RM2Target database to identify the targets of *FTO* and *ALKBH5*. After that, we assessed their expression levels in our RNA-seq data from the ccRCC cohort and compared them to the controls. We found a list of 158 genes encoding the target RNAs of FTO and ALKBH5 in the RM2Target database. Among these genes, 101 genes were differentially expressed in ccRCC patients in our cohort compared to controls (Figure 5), suggesting that they could be potential targets of FTO and ALKBH5 in our cohort. Fourteen genes were upregulated and eighty-seven genes were downregulated in ccRCC patients compared to controls. According to the RM2Target database, 58 genes encode RNAs targeted by FTO, 34 genes encode RNAs targeted by ALKBH5, and 10 genes encode RNAs targeted by both ALKBH5 and FTO. Out of the 101 differentially expressed target genes, eight genes—peroxisome proliferator-activated receptor gamma coactivator 1-alpha (*PGC1A*)*,* metastasis-associated lung adenocarcinoma transcript 1 (*MALAT1*), solute carrier family 1 member 5 (*SLC1A5*), bromodomain containing 9 (*BRD9*), nuclear respiratory factor 1 (*NRF1*), mitochondrial transcription factor A (*TFAM*), cytochrome C oxidase subunit (*COX5A*), and peroxisome proliferator-activated receptor alpha (*PPARA*)—have been identified as targets in kidney cell lines. In our cohort, *MALAT1* (log_2_FC = −4.6) is a downregulated target of ALKBH5. Genes of FTO targets include *PGC1A* (log_2_FC = −2.7)*, COX5A* (log_2_FC = −3.7), *NRF1* (log_2_FC = −3.8), *TFAM* (log_2_FC = −2.3), *SLC1A5* (log_2_FC = −3.4), *PPARA*, (log_2_FC = −4.6), and *BRD9* (log_2_FC = −5.5).

## 4. Discussion

Recent studies have increasingly revealed that the regulation of RMPs is disrupted in cancer. Aberrations in RMPs have been shown to play critical roles in tumorigenesis, affecting cell proliferation, migration, invasion, tumor drug resistance, and the response to immunotherapy [44]. FTO and ALKBH5 are members of the AlkB homolog (ALKBH) family. They catalyze RNA demethylation through an oxidation reaction that involves the conversion of α-ketoglutarate to succinate, along with the release of formaldehyde and carbon dioxide [13,45]. In ccRCC, FTO and ALKBH5 have been found to interact with RCC pathways. The Von Hippel–Lindau (*VHL*) gene is the most commonly mutated gene in ccRCC. In ccRCC, the loss of VHL leads to the accumulation of hypoxia-inducible factor (HIF) protein, which activates signaling pathways that promote tumor progression [46]. A synthetic lethal interaction has been detected between FTO and VHL, leading to the reduced growth of VHL-deficient cells both *in vitro* and *in vivo* [20]. Moreover, the expression of ALKBH5 was induced by HIF under hypoxic conditions, promoting RCC cell proliferation [22]. The expression of FTO and ALKBH5 has shown discrepancies in studies on RCC patients, with some indicating oncogenic roles [19,20,21,22] and others suggesting tumor-suppressive roles [23,24]. Given the limited research focusing on patients from this region, we aimed to perform genotyping and assess the expression of FTO and ALKBH5 in an RCC cohort from the MENA region.

Although the vast majority of somatic mutations in cancer occur in non-coding regions, fewer non-coding variants have been identified compared to coding variants [47]. Non-coding genetic variants in cancer can alter protein expression through different mechanisms. For example, allele-specific transcriptional regulation occurs due to the differential binding of transcription factors as a result of the presence of a genetic variant in one of the two copies of the alleles. This can lead to allelic imbalance when one copy of the alleles is significantly more expressed than the other [48]. In addition, non-coding genetic variants in cancer might disrupt chromatin loops and alter the binding of transcription factors [49]. Another mechanism by which genetic variants alter gene expression is through altering microRNA binding sites, which can disrupt the biological pathways implicated in tumor progression [50]. Single-nucleotide polymorphisms (SNPs) in *FTO* and *ALKBH5* are associated with cancer risk [51,52]. In this study, we assessed the association of a group of non-coding variants of *FTO* and *ALKBH5* and the risk of RCC in a cohort from the MENA region. Our results revealed that the risk of ccRCC increased in patients with the rs11075995T variant of *FTO*. Allele A of rs11075995 was associated with the risk of estrogen-negative breast cancer [53,54] and basal-like breast cancer [55]. Although rs11075995 was associated with breast cancer risk, this association was not detected after adjustment for body mass index (BMI) [56]. Therefore, the rs11075995 FTO variant is not associated with breast cancer risk independently of obesity.

In this study, we did not detect any significant association between the risk of RCC and *FTO* variants rs1121980, rs17817449, rs9939609, and rs8050136 or ALKBH5 variants rs8400, rs2047281425, rs61999283, rs17855125, and rs8068517, which are the other SNPs we looked for in this study. In a Brazilian cohort of breast cancer patients and controls, the *FTO* SNPs rs1121980 and rs9939609, in combination with the melanocortin-4 receptor (*MC4R*) SNP rs17782313, were linked with an increased risk of breast cancer by 4.59-fold [57]. In a study conducted on an African-American cohort, the *FTO* SNPs rs17817449 and rs8050136 were associated with colorectal adenomas [58]. However, other studies failed to detect any association between the risk of CRC and rs17817449 in a population of Slavs [59], or rs9939609 in an Italian population [60]. Another study found that the rs9939609 SNP was not associated with cancer risk unless it was adjusted for BMI in East Asian and African populations [56]. In a Chinese population, the *ALKBH5* variant rs8400 was found to be associated with an increased neuroblastoma risk [61]. However, there was no significant association of rs8400 with the risk of Wilms tumors in Chinese children [62].

In this study, the levels of the FTO protein were significantly lower in ccRCC (*p* < 0.05) and chRCC (*p* < 0.01) patients compared to controls. However, no significant difference was observed in pRCC, similar to ALKBH5. The reduced expression of FTO and ALKBH5 in ccRCC and chRCC, but not in pRCC, is intriguing. One possible explanation for this observation is the presence of different genetic profiles among these subtypes. This variation may influence the signaling pathways and molecular features of each subtype. A similar finding was reported by Strick et al. (2020) [24], who observed that the expression of FTO was significantly higher in pRCC than in both ccRCC and chRCC. Additionally, ALKBH5 levels were significantly higher in pRCC compared to ccRCC, although no significant difference was found when comparing it with chRCC [24]. A study on ccRCC patients in China reported lower expression levels of FTO [23]. In contrast, other studies conducted in the United States [20] and China [21] indicated an increased expression of FTO in ccRCC patients. In this study, the ALKBH5 protein was significantly downregulated in ccRCC (*p* < 0.001) and chRCC (*p* < 0.001) patients compared to controls, with no significant difference observed in pRCC patients. A similar trend was noted in a ccRCC patient cohort from a study conducted in Germany [24]. Conversely, ALKBH5 was found to be upregulated in an RCC cohort from a study conducted in China [22]. Therefore, the expression levels of FTO and ALKBH5 in RCC are heterogeneous and vary among different populations.

This study tested the correlation between FTO protein expression and rs1107997T variants. We found a significant negative correlation (*p* = 0.032) between FTO protein expression and mutated allele frequency at rs11075995T. This indicates a potential role of rs11075995 in regulating FTO expression. In contrast, a study conducted on breast cancer reported a marginally significant reduction (*p* = 0.05) in *FTO* gene expression in the breast tissue of patients with the AA homozygous genotype [54]. These inconsistent results may arise from the use of a different tissue source or genetic variations among different populations.

A significant reduction in *FTO* and *ALKBH5* gene expression was also observed in our cohort at the RNA level assessed by transcriptomic analysis (log_2_FC = −5.2, *q*-value < 0.001, and log_2_FC = −4.7, *q*-value < 0.001, respectively) and RT-qPCR (*p* < 0.01, and *p* < 0.01, respectively) in ccRCC patients relative to controls. To identify the potential targets of the FTO and ALKBH5 proteins in our cohort, we retrieved the FTO and ALKBH5 targets reported in the RM2Target database based on validated results obtained from *in vitro* studies on different cell lines. Among the retrieved targets of FTO and/or ALKBH5, we found 101 differentially expressed target genes in our ccRCC cohort relative to controls. Among them, previous studies validated eight genes in kidney cell lines. In 769-P renal cell carcinoma cells, overexpression of *FTO* leads to the upregulation of *PGC1A*, *TFAM,* and *NRF1* genes, which are involved in mitochondrial biogenesis. Additionally, it induced the expression of the *COX5A* gene, which is involved in oxidative phosphorylation. The induction of mitochondrial biogenesis and oxidative phosphorylation was associated with tumor suppression [23]. In agreement with the previous study, our study revealed that these genes were significantly downregulated in ccRCC patients with downregulated FTO expression relative to controls. In Caki-2 RCC cell lines, *BRD9* was identified as an FTO target and was downregulated upon *FTO* knockdown [21]. In this study, *BRD9* was significantly downregulated in ccRCC patients relative to controls. In VHL-deficient RCC cell lines, the *SLC1A5* gene was significantly downregulated upon *FTO* knockdown [20], consistent with the downregulation of the *SLC1A5* gene in ccRCC patients in our cohort.

In the human kidney cell line HK-2, silencing *ALKBH5* resulted in the downregulation of *MALAT1* [63]. In our study, *MALAT1* was significantly downregulated in ccRCC patients compared to controls. Furthermore, in HK-2 cells, FTO targeted the mRNA of *PPARA*. Overexpression of FTO caused the downregulation of *PPARA* [64]. Conversely, we observed a significant downregulation of *PPARA* in ccRCC patients, who exhibited a significant downregulation of *FTO* relative to controls. This could be attributed to differences in genetic backgrounds and the involvement of various targets of FTO.

The limitations of this study include a relatively small cohort size. The prevalence of RCC is comparatively low compared to other cancers, which significantly restricted the number of eligible participants. Expanding the cohort size is essential to validate the results and explore the effects of FTO and ALKBH5 on clinical progression. Another limitation is the lack of BMI measurements for control samples, which are necessary for the adjustments required to assess whether the rs11075995T variant is linked to obesity in RCC patients. Beyond its role in cancer, FTO also contributes to obesity [65]. Therefore, it is crucial to consider obesity status when evaluating whether increased ccRCC risk in patients is associated with FTO SNPs. Nevertheless, this study highlighted the significance of FTO and ALKBH5 in RCC. The findings have the potential to enhance RCC diagnosis. Larger studies are needed to confirm and expand upon our results. Further research is necessary to determine the functional roles of FTO and ALKBH5 and their downstream targets. Additionally, genotyping and expression analysis of FTO and AlkBH5 should be assessed in varying RCC subtypes and diverse populations.

## 5. Conclusions

The heterogeneous nature of FTO and ALKBH5 expression among different populations underscores the importance of assessing their expressions in various populations. The current study investigated the genotype and expression of FTO and ALKBH5 in an RCC cohort composed of ccRCC, chRCC, and pRCC subtypes from the MENA region. FTO and ALKBH5 were downregulated in ccRCC and chRCC subtypes compared to controls. Additionally, the *FTO* variant rs11075995T is associated with an increased risk of ccRCC in our cohort and correlated with decreased FTO protein expression. Moreover, the potential targets of FTO and ALKBH5 in our cohort were reviewed. These findings not only provide a more in-depth understanding of ccRCC molecular heterogeneity but also have the potential to aid in patient stratification and serve as a foundation for future diagnostic and prognostic tools in RCC. However, further studies are required to confirm our results on larger RCC patient cohorts, investigate the clinical implications, and perform functional validation of the potential targets of FTO and ALKBH5. Additionally, the role of *FTO* variant rs11075995T on its expression needs to be investigated via functional experiments such as dual luciferase reporter assays, chromatin immunoprecipitation, and electrophoretic mobility shift assays. Additionally, further research, including loss of function or gain of function studies, would be necessary to clarify whether the downregulation of FTO and ALKBH5 is a consequence or a driver of tumorigenesis. To our knowledge, this study is the first to investigate the genotype and expression of FTO and ALKBH5 in patients from the MENA region. Further studies are needed to reveal their expression patterns in other populations.

## Figures and Tables

**Table 1:** Characteristics of RCC patients. Age is represented as the median, followed by the IQR, while other parameters are represented as *n* (%). RCC, renal cell carcinoma; ccRCC, clear-cell RCC; chRCC, chromophobe RCC; pRCC, papillary RCC; IQR, interquartile range.

Characteristic	ccRCC, *n* = 39	chRCC, *n* = 7	pRCC, *n* = 8
**Age**	55 (49, 62)	57 (51, 60)	62 (58, 70)
Unknown	1	0	0
**Sex**			
Female	12 (31%)	4 (57%)	3 (38%)
Male	26 (67%)	3 (43%)	5 (62%)
Unknown	1 (2.6%)	0	0
**Obesity**			
Underweight	4 (10%)	0 (0%)	0 (0%)
Normal weight	21 (54%)	6 (86%)	6 (75%)
Overweight	10 (26%)	1 (14%)	1 (12%)
Obese	4 (10%)	0 (0%)	1 (12%)
**Diabetes**	11 (28%)	3 (43%)	4 (50%)
**Nuclear grade**			
1	3 (7.7%)	0	0
2	13 (33%)	0	3 (38%)
3	15 (38%)	0	4 (50%)
4	8 (21%)	0	1 (12%)
Not applicable ^1^	0	7	0
**Capsular invasion**			
Negative	28 (72%)	3 (43%)	7 (88%)
Positive	11 (28%)	4 (57%)	1 (12%)
**Renal sinus invasion**			
Negative	31 (79%)	5 (71%)	5 (62%)
Positive	8 (21%)	2 (29%)	3 (38%)
**Extent**			
Localized	27 (69%)	7 (100%)	7 (88%)
Metastatic	12 (31%)	0	1 (12%)
**Tumor stage**			
I	12 (31%)	2 (29%)	3 (38%)
II	10 (26%)	1 (14%)	2 (25%)
III	8 (21%)	4 (57%)	2 (25%)
IV	9 (23%)	0 (0%)	1 (12%)
**Systemic treatment**	11 (28%)	0	3 (38%)
**Status**			
Alive with disease	5 (13%)	0	0
Died of disease	9 (23%)	0	1 (12%)
Cured	25 (64%)	7 (100%)	7 (88%)

^1^ Based on the World Health Organization’s recommendations, the nuclear grade is not applicable for chRCC due to the lack of visible nucleoli in most cases, the presence of innate nuclear pleomorphism, multinucleation, and hyperchromasia [25].

**Table 2:** Characteristics of controls. Age is represented as the median, followed by the interquartile range, while other parameters are represented as *n* (%).

Characteristic	*n* = 11
**Age**	50 (42, 60)
Unknown	3
**Sex**	
Female	6 (86%)
Male	1 (14%)
Unknown	4
**Diagnosis**	
Hydronephrosis	1 (9.1%)
Mild lymphocytic infiltration	1 (9.1%)
Normal	2 (18%)
Pyelonephritis	7 (64%)
**Nationality**	
Egypt	8 (80%)
Iraq	1 (10%)
United Arab Emirates	1 (10%)
Unknown	1

**Table 3:** Association between *FTO* and ALKBH5 gene polymorphisms and renal cell carcinoma susceptibility. Significant odds ratios are shown in bold. The total number of controls or cases is represented by *n*. SNP, single-nucleotide polymorphism; HWE, Hardy–Weinberg equilibrium; OR, odds ratio; CI, confidence interval; ccRCC, clear-cell renal cell carcinoma; pRCC, papillary renal cell carcinoma; chRCC, chromophobe renal cell carcinoma; UTR, untranslated region.

Gene	SNP	SNP Position *	Controls (HWE *p*-Value)	Subtype (Cases)	OR	95% CI	*p*-Value ***
*FTO*	rs1121980 (G>A)	16:53809247 Intron	*n* = 11 (0.001)	ccRCC (*n* = 22)	1.00	(0.36, 2.78)	1.00
				pRCC (*n* = 7)	1.00	(0.26, 3.82)	1.00
				chRCC (*n* = 6)	1.00	(0.25, 4.08)	1.00
	rs17817449 (T>G)	16:53813367 Intron	*n* = 10 (0.43)	ccRCC (*n* = 19)	0.875	(0.288, 2.658)	1.00
				pRCC (*n* = 6)	0.3	(0.052, 1.747)	0.248
				chRCC (*n* = 5)	0.167	(0.018, 1.583)	0.204
	rs9939609 (T>A)	16:53820527 Intron	*n* = 11 (0.87)	ccRCC (*n* = 20)	1.11	(0.095, 12.92)	1.00
				pRCC (*n*= 6)	1.74	(0.07, 46.15) **	1.00
				chRCC (*n* = 6)	1.74	(0.07, 46.15) **	1.00
	rs8050136 (C>A)	16:53816275 Intron	*n* = 11 (0.058)	ccRCC (*n* = 20)	0.942	(0.32, 2.79)	1.00
				pRCC (*n* = 7)	0.7	(0.164, 2.981)	0.73
				chRCC (*n* = 6)	0.875	(0.199, 3.85)	1.00
	rs11075995 (A>T)	16:53855291 Intron	*n* = 11 (0.38)	ccRCC (*n* = 22)	**3.24**	**(1.06, 9.87)**	**0.046**
				pRCC (*n*= 7)	2.08	(0.5, 8.7)	0.49
				chRCC (*n* = 6)	1.67	(0.39, 7.2)	0.72
*ALKBH5*	rs8400 (G>A)	17:18112845 3′ UTR	*n* = 9 (2.56)	ccRCC (*n*=15)	2.68	(0.71, 10.07)	0.214
				pRCC (*n*= 4)	5.83	(0.95, 35.72)	0.078
				chRCC (*n* = 4)	5.83	(0.95, 35.72)	0.078

* Genomic position according to hg19 (GRCh37). ** Corrected by Haldane–Anscombe correction. *** Two-sided *p*-value based on Fisher’s exact test.

## Data Availability

The RNA-seq data generated in this study have been deposited in GEO under accession number GSE285469 (https://www.ncbi.nlm.nih.gov/geo/query/acc.cgi?acc=GSE285469, accessed on 20 April 2025). All other supporting data are available on request from the corresponding author.

## Supplementary Materials

The following supporting information can be downloaded at: https://www.mdpi.com/article/10.3390/cancers17091395/s1, Table S1: Summary table outlining the samples used in each experimental approach; Table S2: Sequences of tagged primers targeting *FTO* variants; Table S3: Sequences and efficiencies of RT-qPCR primers.

## Abbreviations

The following abbreviations are used in this manuscript:

ALKBH	AlkB homolog
ANOVA	Analysis of variance
BAM	Binary alignment map
ccRCC	Clear cell renal cell carcinoma
chRCC	Chromophobe renal cell carcinoma
CI	Confidence interval
CRC	Colorectal cancer
DAB	3,3′-Diaminobenzidine
DEG	Differentially expressed gene
EMT	Epithelial-mesenchymal transition
FDR	False discovery rate
FFPE	Formalin-fixed paraffin-embedded
HWE	Hardy-Weinberg equilibrium
IFC	Integrated fluidic circuit
IGV	Integrative Genomics Viewer
IHC	Immunohistochemistry
IQR	Interquartile range
IRS	Immunoreactive score
MENA	Middle East and Northern Africa
NGS	Next generation sequencing
NSCLC	Non-small-cell lung cancer
OD	Optical density
OR	Odds ratio
PC	Principal component
PCA	Principal component analysis
PD-L1	Programmed death-ligand 1
pRCC	Papillary renal cell carcinoma
RCC	Renal cell carcinoma
RMP	RNA-modifying protein
RNA-seq	RNA sequencing
RT-qPCR	Quantitative real-time PCR
SNP	Single-nucleotide polymorphism
TMAP	Torrent Mapping Alignment Program
UTR	Untranslated region
