# Supplementary material for: Analysis of Genotype and Expression of FTO and ALKBH5 in a MENA-Region Renal Cell Carcinoma Cohort
Source: Cancers (Basel). 2025 Apr 22;17(9):1395. doi: 10.3390/cancers17091395 (PMC12070863; doi:10.3390/cancers17091395)
Supplement: Supplementary file 1 [file cancers-17-01395-s001.zip › cancers-3576769-supplementary.pdf]

Table S1. Summary table outlining which samples were used in each experimental approach.

Sample ID	Diagnosis	Targeted DNA sequencing	IHC	RNA-seq	RT-qPCR
ccRCC_1	ccRCC	NA	✓	NA	NA
ccRCC_2	ccRCC	✓	✓	NA	NA
ccRCC_3	ccRCC	✓	✓	✓	✓
ccRCC_4	ccRCC	NA	✓	NA	NA
ccRCC_5	ccRCC	✓	✓	NA	NA
ccRCC_6	ccRCC	✓	✓	✓	NA
ccRCC_7	ccRCC	NA	✓	✓	NA
ccRCC_8	ccRCC	✓	✓	NA	NA
ccRCC_9	ccRCC	✓	✓	✓	NA
ccRCC_10	ccRCC	NA	✓	✓	NA
ccRCC_11	ccRCC	NA	✓	NA	NA
ccRCC_12	ccRCC	✓	✓	NA	NA
ccRCC_13	ccRCC	✓	✓	NA	✓
ccRCC_14	ccRCC	NA	✓	NA	NA
ccRCC_15	ccRCC	✓	✓	NA	NA
ccRCC_16	ccRCC	NA	✓	NA	NA
ccRCC_17	ccRCC	✓	✓	✓	NA
ccRCC_18	ccRCC	✓	✓	NA	NA
ccRCC_19	ccRCC	NA	✓	✓	NA
ccRCC_20	ccRCC	NA	✓	NA	NA
ccRCC_21	ccRCC	✓	✓	NA	✓
ccRCC_22	ccRCC	✓	✓	NA	✓
ccRCC_23	ccRCC	✓	✓	✓	NA
ccRCC_24	ccRCC	✓	✓	NA	✓
ccRCC_25	ccRCC	NA	✓	NA	NA
ccRCC_26	ccRCC	✓	✓	✓	NA
ccRCC_27	ccRCC	✓	✓	NA	NA
ccRCC_28	ccRCC	✓	✓	✓	NA
ccRCC_29	ccRCC	NA	✓	✓	✓
ccRCC_30	ccRCC	✓	✓	✓	NA
ccRCC_31	ccRCC	✓	✓	✓	✓
ccRCC_32	ccRCC	✓	✓	NA	✓

ccRCC_33	ccRCC	NA	✓	NA	NA
ccRCC_34	ccRCC	✓	✓	NA	NA
ccRCC_35	ccRCC	NA	✓	NA	NA
ccRCC_36	ccRCC	NA	✓	NA	NA
ccRCC_37	ccRCC	NA	✓	NA	NA
ccRCC_38	ccRCC	NA	✓	NA	NA
ccRCC_39	ccRCC	NA	✓	NA	NA
chRCC_1	chRCC	✓	✓	NA	NA
chRCC_2	chRCC	✓	✓	NA	NA
chRCC_3	chRCC	✓	✓	NA	NA
chRCC_4	chRCC	✓	✓	NA	NA
chRCC_5	chRCC	NA	✓	NA	NA
chRCC_6	chRCC	✓	✓	NA	NA
chRCC_7	chRCC	✓	✓	NA	NA
pRCC_1	pRCC	✓	✓	NA	NA
pRCC_2	pRCC	✓	✓	NA	NA
pRCC_3	pRCC	✓	✓	NA	NA
pRCC_4	pRCC	NA	✓	NA	NA
pRCC_5	pRCC	✓	✓	NA	NA
pRCC_6	pRCC	✓	✓	NA	NA
pRCC_7	pRCC	✓	✓	NA	NA
pRCC_8	pRCC	✓	✓	NA	NA
Control 1	control	✓	✓	✓	✓
Control 2	control	✓	✓	NA	NA
Control 3	control	✓	✓	✓	✓
Control 4	control	✓	✓	✓	✓
Control 5	control	✓	✓	✓	NA
Control 6	control	✓	✓	✓	✓
Control 7	control	✓	✓	✓	✓
Control 8	control	✓	✓	✓	✓
Control 9	control	✓	✓	✓	✓
Control 10	control	✓	✓	✓	NA
Control 11	control	✓	✓	✓	✓

Table S2. Sequences of tagged primers targeting *FTO* variants. The primers were attached to Fluidigm-specific tag sequences (shown in uppercase) at the 5′ end of forward and reverse primers.

Gene	Sequence ID	Sequence (5' > 3')	Product length (bp)
FTO	rs9939609	Forward: ACACTGACGACATGGTTCTACAttattctaggttccttgcgact	159
		Reverse: TACGGTAGCAGAGACTTGGTCTtttgcttttatgctctccca	
	rs17817449	Forward: ACACTGACGACATGGTTCTACAtgtgtaactggagtctcccct	199
		Reverse: TACGGTAGCAGAGACTTGGTCTtgggagtgcaccaaattcaa	
	rs8050136	Forward: ACACTGACGACATGGTTCTACAtttccctgggacctgtgaca	175
		Reverse: TACGGTAGCAGAGACTTGGTCTagcattccatgagtccatctct	
	rs1121980	Forward: ACACTGACGACATGGTTCTACAtcttatatggccccacctttc	128
		Reverse: TACGGTAGCAGAGACTTGGTCTaatcaagagttacaggtaggcagg	
	rs11075995	Forward: ACACTGACGACATGGTTCTACActtcttcatcattgctttgagtat	136
		Reverse: TACGGTAGCAGAGACTTGGTCTgccatgagcagcaactctaa	
ALKBH5	rs8068517	Forward: ACACTGACGACATGGTTCTACAtgaacccaggtctcttcaact	163
		Reverse: TACGGTAGCAGAGACTTGGTCTagaacccactgactttgagc	
	rs8400	Forward: ACACTGACGACATGGTTCTACActcctccgcttcagggtatg	164
		Reverse: TACGGTAGCAGAGACTTGGTCTccacaaaaccttgctgcctt	
	rs2047281425	Forward: ACACTGACGACATGGTTCTACAtaacgtcctacctccctttc	187
		Reverse: TACGGTAGCAGAGACTTGGTCTgaggactcgtatgacttgcg	
	rs61999283	Forward: ACACTGACGACATGGTTCTACAtatcaggaggactcggaccc	166
		Reverse: TACGGTAGCAGAGACTTGGTCTcactcgtcctggctgaagag	
	rs17855125	Forward: ACACTGACGACATGGTTCTACAacggatcctggagatggaca	159
		Reverse: TACGGTAGCAGAGACTTGGTCTtcaggactcgtatgacttgcg	

Sequences and efficiency assessment of RT-qPCR primers

The efficiency of *FTO*, *ALKBH5*, and *HMBS* RT-qPCR primers (Table S3) was assessed using 786-O cDNA. The efficiency was evaluated against a range of cDNA concentrations, and each concentration was tested in triplicates. Each RT-qPCR reaction contained 5µl 2X SYBER green, 0.1µl forward primer (100nM), 0.1µl reverse primer (100nM). Depending on the tested cDNA concentration, nuclease-free water and cDNA were added to reach a final volume of 10µl. RT-qPCR reaction was carried out using QuantStudio™ 3 Real-Time PCR System (Applied Biosystems), and the data were analysed by plotting Ct values against the cDNA concentrations at log10 scale. Then, a linear regression curve was generated through the data points and calculate the slope of the trend line. The efficiency is calculated using the following equation:

$$E = 10^{\frac{-1}{slope}} - 1$$

The calculated efficiency percentage fell within the accepted range of 90–110%, indicating reliable and efficient amplification of all primer pairs (Table S3).

Table S3. Sequences and efficiencies of RT-qPCR primers. FTO, fat mass and obesity-associated protein; ALKBH5, alkB homolog 5 RNA demethylase; HMBS, hydroxymethylbilane synthase.

Gene	Primer sequence (5' > 3')	Product length (bp)	Slope	Efficiency	Efficiency%
FTO	Forward: ctgtgttttggccggttcac	103	-3.33	1.00	99.69
	Reverse: agccaactgacagcgttgta				
ALKBH5	Forward: gaccctgctctgaaacccaa	82	-3.20	1.05	105.17
	Reverse: tgtccatctccaggatccgt				
HMBS	Forward: accctcatgatgctgttgtctt	81	-3.15	1.08	107.71
	Reverse: ttcccaccacactcttctctgg				
